# The distinct biological role of JAML positions it as a promising target for treating human cancers and a range of other diseases

**DOI:** 10.3389/fimmu.2025.1558488

**Published:** 2025-06-25

**Authors:** Zhilin Dong, Ning Liu, Meili Sun

**Affiliations:** Department of Oncology, Central Hospital Affiliated to Shandong First Medical University, Jinan, Shandong Province, China

**Keywords:** cancer immunotherapy, junctional adhesion molecule-like protein (JAML), lipid metabolism, T-cell infiltration, inflammatory response

## Abstract

Junctional adhesion molecule-like protein (JAML), a member of the junctional adhesion molecule (JAM) family, is widely expressed in various cells. This review examines the progress made in understanding JAML, beginning with its discovery and subsequent characterization. It emphasizes the mechanisms through which JAML influences biological processes, including cell migration, inflammation, lipid metabolism, and antitumor immunity, as demonstrated by recent studies. In addition, the clinical relevance of JAML is analyzed in the context of diseases linked to these processes. Particular attention is given to the effects of JAML on tumor cell proliferation and migration, alongside its pivotal role in regulating lymphocyte infiltration into the tumor microenvironment. Collectively, The balance between JAML’s pro-inflammatory and anti-tumor functions underscores its therapeutic promise.

## 1 Introduction

Junctional adhesion molecules (JAMs), essential constituents of tight junctions, have emerged as a significant focus of research. JAMs belong to a glycoprotein family within the immunoglobulin superfamily (IgSF) (1), with key members including JAM-A, JAM-B, JAM-C, JAM-4, and JAML. These proteins are pivotal in regulating cell polarity, maintaining epithelial barrier integrity, and facilitating leukocyte trafficking. Structurally, JAMs are characterized by two extracellular immunoglobulin-like domains, a transmembrane region, and a cytoplasmic tail (2–5). The cytoplasmic tail contains a PDZ-binding motif, a protein-protein interaction domain first identified in proteins such as PSD-95, Discs large, and Zona occludens-1, from which it derives its name. PDZ domains are critical for assembling protein complexes by interacting with short peptide motifs at the C-terminus of target proteins (6). Junctional adhesion molecule-like protein (JAML), the newest member of the JAM family, is unique among JAM proteins due to the absence of a conventional PDZ-binding motif at its C-terminus (4).

**Figure 1:**
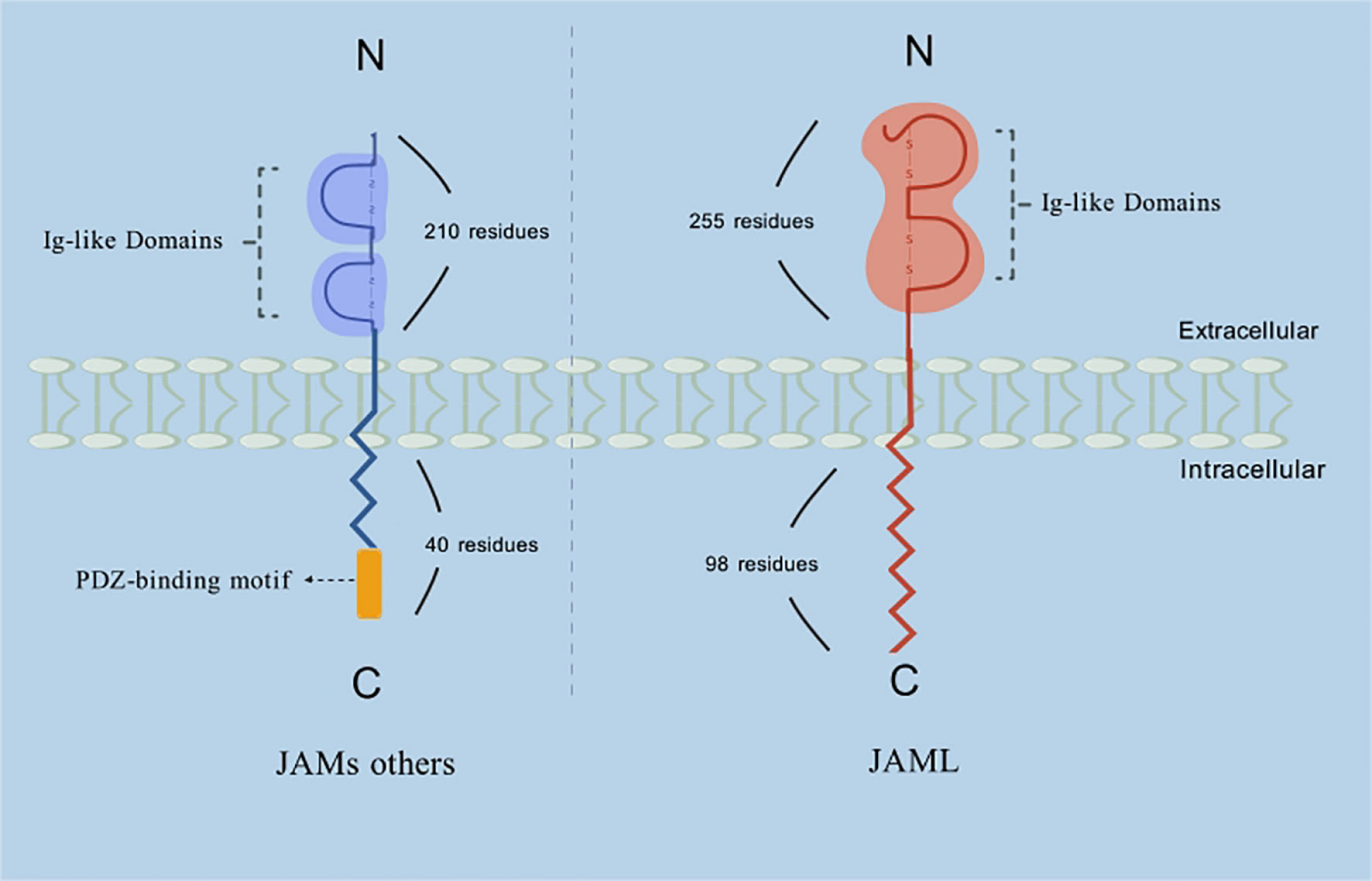
Schematic representation of the structure of JAML and other members of the JAMs family. Description: Among the JAM family, JAML’s extracellular domain is unique due to its considerably larger size (~255 residues compared to ~210 residues in other JAM proteins). However, the functional implications of this size difference remain unclear. Furthermore, while JAM-A, -B, and -C possess relatively short cytoplasmic tails of about 40 amino acids, JAML’s cytoplasmic tail is significantly longer, containing 98 residues.

Compared to other JAM family members, the absence of a PDZ-binding motif in JAML (**Figure 1**) indicates unique possibilities for its protein function and targeting mechanisms. Specifically, JAML may exhibit diverse expression patterns and distinct roles in various diseases. Studies have shown that JAML is expressed in neutrophils, monocytes, tissue-resident γδT cells, activated lymphoid γδT cells, αβT cells, and acute promyelocytic leukemia cells (7). It plays a critical role in mediating the adhesion and migration of immune and endothelial/epithelial cells, thereby contributing to the regulation of inflammatory responses. In recent years, the involvement of JAML in tumor biology has garnered increasing attention. Research reveals that JAML participates not only in tight junctions and innate immune processes related to inflammation but also in adaptive immune responses, underscoring its pivotal role in immune regulation.

This review summarizes the roles of JAML in both tumor and non-tumor diseases, emphasizing its functions in immune-related physiological processes. Furthermore, it aims to provide an overview of the current research landscape on JAML, highlighting its potential strengths and limitations as a novel immunotherapeutic target.

## 2 Main text

### 2.1 The JAM family and junctional adhesion molecule-like protein

Tight junctions (TJs) play a vital role in regulating the migration of extracellular ions, small molecules, and inflammatory cells. They also define distinct membrane domains and enable bidirectional signaling between intracellular and extracellular compartments. Junctional adhesion molecules (JAMs), members of the immunoglobulin superfamily (IgSF) (1), are characterized by distinct structural features, including two IgSF domains: a membrane-distal V-type and a membrane-proximal C2-type Ig domain, as well as a transmembrane domain and a cytoplasmic tail (2–5). Among the JAM family, JAML’s extracellular domain is unique due to its considerably larger size (~255 residues compared to ~210 residues in other JAM proteins). However, the functional implications of this size difference remain unclear. Furthermore, while JAM-A, -B, and -C possess relatively short cytoplasmic tails of about 40 amino acids, JAML’s cytoplasmic tail is significantly longer, containing 98 residues (**Figure 1**) (8).

JAM-A was the first member of the JAM family to be identified, initially discovered as a receptor for a specific antibody. This antibody interacts uniquely with JAM-A, triggering platelet activation, promoting platelet aggregation, increasing granule secretion, and enhancing fibrinogen binding (9). Following this discovery, JAM-B (10) and JAM-C (11) were also cloned and identified, together forming the junctional adhesion molecule (JAM) family. Subsequent research revealed distinct differences in the cellular localization and tissue expression patterns of the three canonical JAM family members: JAM-A, JAM-B, and JAM-C. Among them, JAM-A has the broadest expression profile, being found in tissue endothelial and epithelial cells, as well as in platelets, neutrophils, monocytes, lymphocytes, and erythrocytes (12, 13), In contrast, JAM-B is predominantly expressed in endothelial cells, with tissue-specific expression in the heart, lymph nodes, brain, and kidneys (10), JAM-C, meanwhile, is also expressed in endothelial cells and leukocytes, with additional expression observed in tissues such as the intestine, lymph nodes, testes, liver, placenta, and brain (3, 11).

JAM-A, JAM-B, and JAM-C are critical regulators of cell polarity, epithelial barrier function, and leukocyte migration. JAM-A plays a key role in maintaining the stability of the blood-testis barrier (BTB) (14).In epithelial barriers, it inhibits cell migration and reduces collective cell movement (15); JAM-B, on the other hand, promotes germ cell migration (16), facilitates hematopoietic cell homing, and enhances angiogenesis (17);Meanwhile, JAM-C mediates the polarization and differentiation of sperm cells, leading to the production of mature sperm (18), and inhibits cell migration in epithelial barriers, thereby reducing barrier permeability (19).

In 2003, Christel Moog-Lutz et al. (4) identified junctional adhesion molecule-like protein (JAML) as a novel retinoic acid-induced gene in acute promyelocytic leukemia (APL) cells, considering it a marker of granulocyte differentiation. As a closely related JAM-like molecule, JAML was reported to interact with the coxsackie and adenovirus receptor (CAR), a protein localized in the tight junctions (TJs) of epithelial and certain endothelial cells. Both JAML and CAR are type I glycoproteins, characterized by two extracellular immunoglobulin-like domains, a transmembrane segment, and a cytoplasmic tail. JAML mRNA is expressed in hematopoietic tissues, with particularly high levels in granulocytes. The JAML protein has been found in neutrophils, monocytes, macrophages, tissue-resident γδ T cells, activated lymphoid γδ T cells, αβ T cells, renal podocytes, tumor cells, and APL cells (7). Notably, JAML localizes to the plasma membrane at cell-cell contact regions but is absent at free cell boundaries (4), suggesting its involvement in homotypic interactions and a potential role in leukocyte trafficking. In recent years, JAML, the most recently discovered member of the JAM family, has gained increasing attention for its roles in lipid metabolism disorders, inflammatory responses, tumor cell proliferation and migration, and antitumor immunity.

### 2.2 The Function of JAML in normal cells and tissues

#### 2.2.1 JAML promotes leukocyte infiltration into inflammatory tissues and exerts pro-inflammatory effects

Veronika Mraz et al. (20) demonstrated the critical role of JAML in contact hypersensitivity using JAML-KO and JAML-WT mice. Their study revealed that during sensitization with contact allergens, JAML-knockout mice exhibited weakened inflammatory responses and reduced recruitment of CD8+ and CD4+ T cells to the epidermis. Moreover, normal JAML expression was found to be essential for the effector function of CD8+ TRM cells and the production of IFNγ. In summary, JAML is indispensable for T-cell infiltration into the epidermis and the effector function of CD8+ TRM cells. Multiple sclerosis (MS), an immune-mediated disease of the central nervous system (CNS), is characterized by multifocal leukocyte infiltration, demyelination, and axonal damage. In studies investigating leukocyte migration to the CNS during MS pathogenesis, Jorge Ivan Alvarez et al. (21) observed significant upregulation of JAML in the blood-brain barrier, monocytes, and CD8+ T cells of MS patients. These findings were derived from *in vitro*, ex vivo, and postmortem human samples. Notably, blocking JAML significantly impaired leukocyte migration. The blood-brain barrier (BBB) plays a crucial role in cell migration. as part of its active role supporting leukocyte transmigration, the BBB projects transmigratory cups rich in actin and CAMs that anchor and embrace adherent leukocytes (22). JAML, as a member of CAMs, has been shown to be enriched in transmigratory cups (21). Analysis of junctional molecules supporting BBB function such as p120 demonstrated localization at inter-endothelial junctions and in docking structures supporting adherent monocytes. Transmigratory cup-like structures expressing JAML were also detected in areas of contact between CD8T cells and the BBB. These studies demonstrate that JAML plays a crucial role in mediating the migration of monocytes and CD8 T cells across the blood-brain barrier. As is well known, the number and function of CD8 T cell infiltration in the tumor microenvironment are critical factors influencing tumor prognosis, with tumors exhibiting immune desert generally having a worse outcome. Currently, treatment for brain tumors is primarily surgical. However, due to the blood-brain barrier, drugs often fail to achieve significant therapeutic efficacy, and tumor-killing cells, such as CD8 T cells, struggle to infiltrate the tumor microenvironment. Recent evidence has shown that JAML is present in the blood-brain barrier and plays a key role in mediating the migration of CD8 T cells. Targeting JAML may offer a promising new approach for treating brain tumors. Additionally, as a ligand of CAR, JAML interacts with CAR, which is typically localized at epithelial tight junctions but is diffusely expressed in the choroid plexus (23). During relapsing-remitting multiple sclerosis (RRMS), JAML is proposed to mediate CNS leukocyte migration through heterotypic interactions with CAR in the choroid epithelial cells of the blood-brain barrier. In the context of atherosclerosis, Ya-Lan Guo et al. (24)investigated the mechanism of early monocyte transendothelial migration (TEM). Their study highlighted the critical role of JAML expressed on monocytes in regulating neutrophil TEM. This process is mediated through interactions with endothelial CAR and other tight junction-associated adhesion molecules, suggesting JAML’s importance in inflammatory cell trafficking during disease progression.

**Figure 2:**
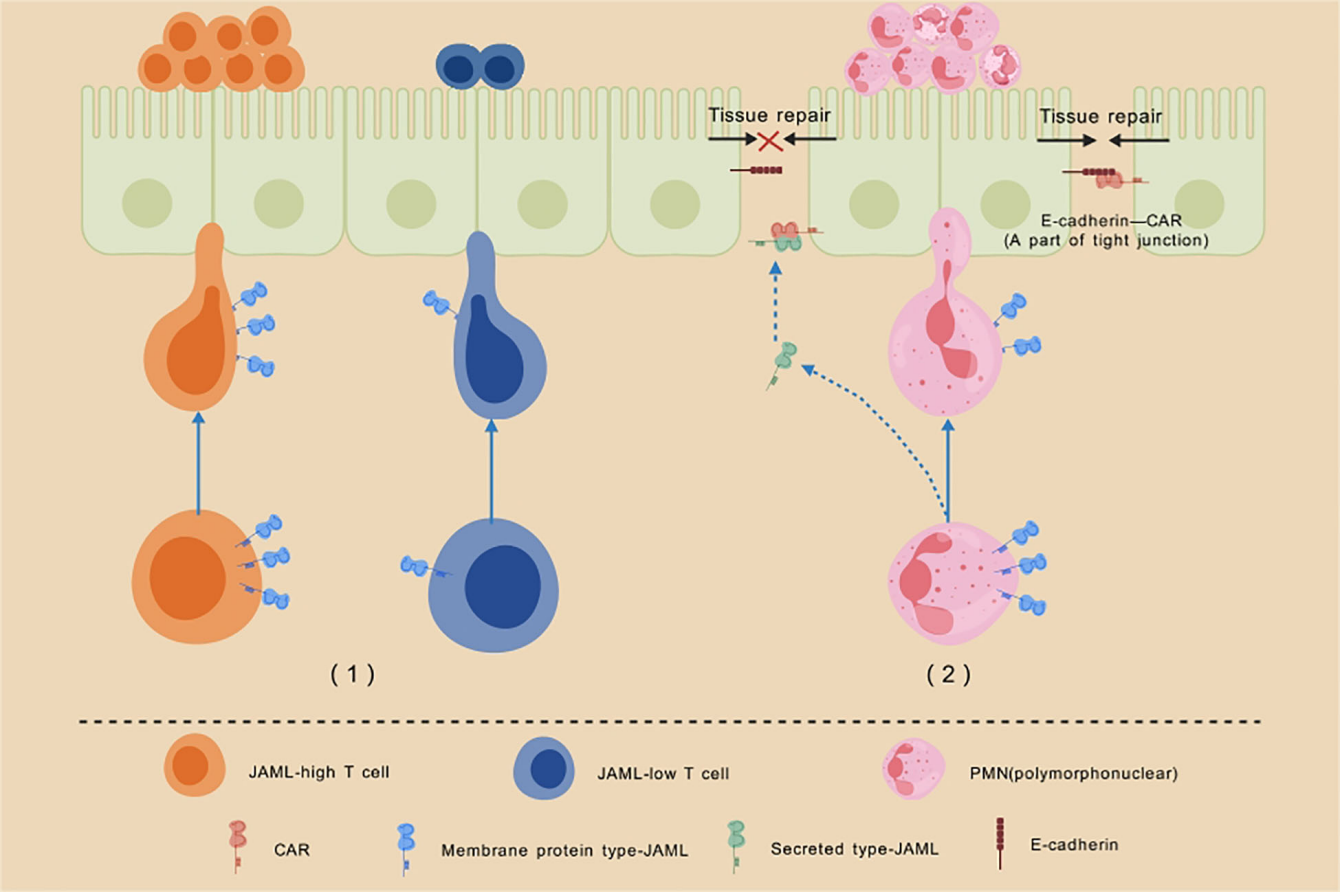
Schematic illustration of the role of JAML in cell chemotaxis and inflammatory response. Description: (1)T cells with high JAML expression demonstrate enhanced recruitment to the epidermis compared to those with low JAML expression. Moreover, normal JAML expression is critical for the effector function of CD8+ TRM cells and the production of IFNγ, highlighting its indispensable role in epidermal immune responses. (2) During the activation, adhesion, and migration of PMNs across the intestinal epithelium, JAML is shed from the cell surface. The secreted JAML released by PMNs binds to CAR on the intestinal epithelial surface, disrupting CAR’s interaction with other adhesion molecules. This interference inhibits intestinal epithelial repair, significantly delaying the healing of inflammatory wounds.

In addition to its role as a membrane protein, JAML also functions as a secreted protein. In a study on acute inflammation by Dominique A. Weber et al. (25), many acute mucosal surface inflammations were found to be characterized by massive infiltration of neutrophils or polymorphonuclear leukocytes (PMNs). The trans-homodimerization of the CAR-D1 domain of the intestinal epithelial cell membrane receptor CAR with CAR on adjacent cells, as well as its interactions with adhesion molecules such as Zonula Occludens 1 (ZO-1), E-cadherin, and β-catenin (26–28), are essential for maintaining cell-cell adhesion and epithelial barrier integrity. During the activation, adhesion, and migration of PMNs across the intestinal epithelium, JAML is shed from the cell surface. The secreted JAML, released by PMNs, binds to CAR on the intestinal epithelial surface, disrupting the interaction between CAR and its ligands. This interference inhibits intestinal epithelial repair, prolonging the inflammatory response. Notably, blocking the interaction between JAML and CAR using anti-JAML monoclonal antibodies has been shown to significantly improve inflammation resolution and epithelial healing (**Figure 2**).

#### 2.2.2 JAML promotes macrophage M1 polarization and exerts pro-inflammatory effects

In a study on acute kidney injury (AKI) by Wei Huang et al. (29), JAML-mediated macrophage polarization was identified as a critical signaling pathway linking inflammation to AKI. Using flow cytometry, the polarization states of two macrophage subtypes, F4/80lo (infiltrating) and F4/80hi (resident), in mouse kidneys were analyzed. The results showed that in Jaml -/- mice, the proportion of M2 macrophages (CD206hi) was significantly higher, while the proportion of M1 macrophages (CD80hi) was significantly lower compared to control mice. This indicates that JAML deficiency promotes M2 macrophage polarization. Macrophages, being highly heterogeneous, differentiate into M1 or M2 phenotypes based on the microenvironment and the disease’s nature or stage. Typically, M1 macrophages exert pro-inflammatory effects in inflammatory responses, while M2 macrophages play anti-inflammatory roles (30).

Atherosclerosis, a chronic inflammatory disease, is a leading cause of heart attacks and strokes. Its development is driven by the infiltration of leukocytes (including monocytes and neutrophils) into the arterial wall, monocyte differentiation into macrophages, and macrophage uptake of oxidized low-density lipoprotein (OxLDL). The activation of macrophages and production of inflammatory cytokines are major contributors to chronic inflammation and plaque progression. In a study by Yu Sun et al. (31), JAML was found to be strongly expressed in atherosclerotic plaques of cardiovascular patients, with immunofluorescence co-staining showing its predominant expression in macrophages. Animal experiments further demonstrated that silencing JAML in mice significantly reduced atherosclerotic lesion formation, decreased necrotic core size, increased plaque fibrous cap thickness, and reduced macrophage content and inflammation. Moreover, JAML was shown to enhance OxLDL-induced macrophage inflammatory cytokine production. These findings suggest that therapeutic strategies targeting JAML function may represent a promising approach to alleviating atherosclerosis and improving plaque stability.

#### 2.2.3 JAML is overexpressed in podocytes of diabetic nephropathy, contributing to lipid metabolic disorders

The glomerulus serves as a size-selective filter in the kidney, removing toxic compounds from the bloodstream. Glomerular diseases, whether primary or secondary to systemic conditions such as diabetes, are significant contributors to chronic kidney disease (CKD). Among the glomerular components, podocytes—terminally differentiated cells critical to the glomerular filtration barrier—are particularly vulnerable to lipotoxicity (32). Dysregulated lipid metabolism, resulting in cholesterol accumulation or increased intracellular free fatty acids, has been shown to induce lipotoxic injury in podocytes (33). Lipid accumulation in podocytes is a key pathological factor in diabetic kidney disease (DKD).In a study by Yi Fu et al. (34), elevated JAML expression was observed in the glomeruli and serum of experimental DKD models, DKD patients, and individuals with focal segmental glomerulosclerosis (FSGS) and membranous nephropathy (MN). JAML expression was positively correlated with serum creatinine levels and lipid accumulation in DKD patients, and negatively correlated with their glomerular filtration rate (GFR). Furthermore, podocyte-specific deletion of Jaml significantly improved podocyte injury, reduced proteinuria, mitigated lipid accumulation, and restored kidney function in two DKD mouse models (streptozotocin-fed high-fat diet mice and db/db mice).Additional studies uncovered a novel function of JAML in regulating podocyte lipid metabolism through the Sirt1-mediated SREBP1 signaling pathway, where Sirt1 acts as a key mediator linking JAML to AMPK/SREBP1 signaling. This research established a critical connection between JAML and lipid metabolism, identifying JAML as a novel driver of glomerular disease progression through its role in podocyte lipid metabolism. These findings underscore the therapeutic potential of targeting JAML-mediated lipid metabolism as a strategy for treating both metabolic and non-metabolic glomerular diseases. The role of JAML in lipid metabolism may also extend to other diseases, including atherosclerosis, Alzheimer’s disease, Parkinson’s disease, cardiovascular diseases, and age-related macular degeneration. Further exploration of JAML’s regulatory function in lipid metabolism across these conditions could provide new insights into its broader therapeutic implications.

### 2.3 The function of JAML in cancer

#### 2.3.1 JAML’s dual role: immune activation vs. tumor promotion

Beyond its roles in normal cells, tissues, and certain non-cancerous diseases, JAML’s involvement in tumors has garnered significant interest. Studies on JAML in cancer reveal its dual role (**Table 1**), which depends on whether it is expressed in immune cells or tumor cells. When highly expressed in T lymphocytes, JAML enhances immune responses and exerts anti-tumor effects by boosting the tumor-killing capabilities of T lymphocytes within the tumor microenvironment (TME). Conversely, elevated JAML expression in tumor cells may contribute to tumor progression, underscoring the complex and context-dependent roles of JAML in cancer biology.

**Table 1:** JAML-associated mechanisms in tumor promotion/inhibition and their clinical applications.

Mechanism	References	Effect	Potential application value
1. JAML interacts with CXADR to promote γδT cell and CD8^+^ T cell activation and maintain activated cell activity	(7, 35, 36, 41)	Inhibitory effects on cancers	A. More efficient delivery of DC vaccine to local LNS B. A new combination regimen: agonist anti-JAML antibody and PD-1 were used in combination
2. JAML promotes TLR1/2 expression on CD8^+^T TRMs (tissue-resident CD8^+^T cells)	(42)		
1. Activation of Wnt/ß-catenin signaling pathway in cancer cells to promote EMT	(45)	Cancer-promoting effects	A. Targeting JAML may reverse resistance to immune checkpoint inhibitors in patients with malignancies
2. Activation of p38 signaling pathway to promote cancer cells migration and proliferation	(44)		
3. Activation of PI3K-AKT-mTOR signaling pathway to promote cancer development	(46, 47)		
4. It affects chemokine secretion to regulate T cells infiltration	(47)		

#### 2.3.2 JAML overexpression in T lymphocytes enhances T cell killing functions and suppresses tumor growth

In skin and mucosal tissues, γδ T cells play a crucial role in maintaining epithelial stability and responding to tissue injury by recognizing self-stress molecules independently of MHC antigen presentation. Among various leukocyte populations, higher γδ T cell tumor infiltration serves as the strongest prognostic indicator of improved survival outcomes in cancer patients, highlighting their critical role in anti-tumor immunity (35). Deborah A. Witherden et al. (7)identified a co-stimulatory molecule specific to epithelial γδ T cells, termed junctional adhesion molecule-like protein (JAML). By binding to its ligand, the coxsackie and adenovirus receptor (CAR), JAML provides co-stimulatory signals that enhance γδ T cell proliferation, activation, and the secretion of cytokines and growth factors. Blocking JAML-mediated co-stimulation diminishes γδ T cell activation, mimicking the functional deficiency of these cells. As an early source of tumor-derived IFNγ, γδ T cells act as a critical link between the innate and adaptive immune systems (36).

Dendritic cells (DCs), as professional antigen-presenting cells (APCs), are critical for inducing adaptive immunity and maintaining immune tolerance (37). Under pro-inflammatory conditions or stimulation by pathogen- or damage-associated molecular patterns, DCs capture antigens and migrate to regional lymph nodes (LNs), where they activate naïve or antigen-specific T cells, thereby enabling immune cytotoxicity (38). Over the past decades, significant research efforts have focused on enhancing the efficacy of DC-based cancer immunotherapy. A key strategy has been the targeted delivery of DC vaccines to local LNs to elicit robust anti-tumor immunity (39). However, the molecular mechanisms underlying the transendothelial migration (TEM) of DC vaccines across endothelial barriers to LNs remain poorly understood. Recent findings by Seung-Eon Roh et al. (40)demonstrated that JAML plays a crucial role in facilitating TEM of mouse bone marrow-derived dendritic cells (BMDCs). Blocking JAML using antibodies or knocking out JAML significantly impaired the TEM activity of BMDCs, reducing the efficacy of DC-based cancer immunotherapy. These results highlight JAML, a member of the JAM family, as a key facilitator of TEM in both mouse and human DCs, underscoring its importance in enabling the migration of DCs from inoculation sites to regional LNs during immunotherapy.

JAML is also expressed on activated CD8+ T cells and tissue-resident γδ T cells, where it plays an essential role in sustaining their physiological functions. Clinically, elevated JAML expression on CD8+ T cells and γδ T cells correlates with improved patient survival outcomes. High immunogenic JAML expression limits tumor growth and enhances tumoricidal activity. This effect is mediated by the interaction between JAML and tumor-derived CXADR, which delivers co-stimulatory signals to activate naïve γδ T and CD8+ T cells while maintaining the activity of already activated cells (41).Further investigation into the regulation of JAML expression revealed, through a human chromatin interaction map, strong interactions between the JAML promoter and activation-induced intronic cis-regulatory regions near the CD3D promoter, indicating their role in controlling JAML expression. Studies have also shown that JAML activation enhances TLR1/2 expression on tissue-resident CD8+ T cells (CD8+ TRMs), which suppresses tumor progression and prolongs survival in tumor-bearing mice. Immunofluorescence analysis further demonstrated a positive correlation between high expression levels of JAML, TLR1, and TLR2 on CD8+ T cells and overall survival (OS) in lung cancer patients, suggesting CD8+ TRMs and JAML as valuable biomarkers for predicting NSCLC outcomes and as potential targets for immunotherapy (42). Notably, Joseph M. McGrawde and his team (43), using a mouse melanoma model, showed that combining an agonistic anti-JAML antibody with PD-1 therapy was more effective in suppressing tumor growth than either treatment alone. These findings position JAML as a promising therapeutic target for cancer treatment, with the potential to synergize with existing immunotherapies.

#### 2.3.3 High levels of JAML expression in tumor cells drive tumor proliferation

The elevated expression of JAML in tumor tissues has recently garnered significant attention, with studies revealing its role in enhancing tumor cell proliferation and migration, thereby contributing to increased malignancy.

Yuying Fang and colleagues (44)first reported that JAML expression is significantly higher in gastric cancer tissues compared to adjacent normal tissues. Gastric cancer cells overexpressing JAML exhibited enhanced migratory and proliferative abilities via activation of the p38 signaling pathway. Subsequently, Qian Wu et al. (45)demonstrated that JAML overexpression promotes proliferation, migration, and invasion of lung adenocarcinoma (LUAD) cells, whereas JAML knockdown induces cell cycle arrest and triggers apoptosis. These effects were found to be partially mediated by JAML’s activation of the Wnt/β-catenin signaling pathway, which facilitates epithelial-mesenchymal transition (EMT) in LUAD cells. Further research by Qian Wu et al. (46)revealed that JAML deletion inhibits EMT through the inactivation of the PI3K/AKT/mTOR signaling pathway. Interestingly, kaempferol was shown to suppress the proliferation, migration, and invasion of LUAD cells (A549 and H1299) while partially inhibiting EMT via the PI3K/AKT/mTOR pathway. Notably, JAML knockdown reduced kaempferol’s inhibitory effects on LUAD cells, suggesting that kaempferol’s anticancer properties are mediated, at least in part, by targeting JAML.

Yanan Liu and colleagues (47) explored the prognostic significance of JAML in colorectal cancer (CRC) by analyzing tumor samples from 50 CRC patients. Immunohistochemistry revealed that 50% (25/50) of CRC patients exhibited high JAML expression, primarily localized in the cytoplasm and membranes of cancer cells, with minimal expression in stromal immune cells and negligible levels in adjacent normal tissues. The median overall survival of patients with high JAML expression was significantly lower than those with low JAML expression. The PI3K-AKT-mTOR signaling pathway plays a crucial role in cell growth, proliferation, and tissue development from the embryonic stage to adulthood. Evidence has shown that JAML is involved in this pathway, where it can promote the proliferation, migration, and invasion of colorectal cancer cells by activating the PI3K-AKT-mTOR signaling pathway. This could be one of the reasons why JAML contributes to cell growth and proliferation disorders, ultimately leading to tumorigenesis. Intriguingly, high tumor-derived JAML expression in CRC patients was associated with reduced infiltration of CD3+ and CD8+ T lymphocytes, an observation further validated in animal models. To investigate the underlying mechanisms, PCR analysis revealed that JAML knockout significantly upregulated the expression of chemokines such as CCL20 and CXCL9/10/11, which are implicated in T cell recruitment. However, the mechanisms by which JAML’s biological effects influence tissue growth and development disorders remain to be further investigated.

These findings suggest that JAML plays a consistent role across colorectal cancer, gastric cancer, and lung adenocarcinoma, demonstrating the universality of its mechanisms in promoting malignancy. This underscores JAML’s significant potential as a therapeutic target and highlights its substantial value in cancer research.

## 3 Discussion

Junctional adhesion molecule-like protein (JAML), a novel member of the junctional adhesion molecule (JAM) family, is expressed in a diverse range of cell types, including neutrophils, monocytes, macrophages, tissue-resident γδ T cells, activated lymphoid γδ T cells, αβ T cells, kidney podocytes, and solid tumor cells. Analyzing the existing research, it can be observed that JAML is a mediating molecule that facilitates the involvement of immune cells in disease processes. Both secretory and constitutive forms of JAML can bind to its receptor CAR, thereby influencing the migration of T cells and neutrophils across various barriers in the body, which in turn affects the progression of inflammatory responses, multiple sclerosis, and other disease processes. At the same time, JAML regulates macrophage polarization and lipid metabolism in renal podocytes, influencing the onset and progression of acute kidney injury and diabetic nephropathy. Importantly, JAML also plays a critical role in antitumor immunity. Within tumor microenvironments, tumor-derived JAML promotes tumor cell proliferation and migration, facilitates malignant progression, and suppresses T lymphocyte infiltration by downregulating chemokine secretion. This reduction in chemokine levels impairs immune cell recruitment, enabling tumor growth and immune evasion. All of the above indicate the uniqueness of JAML compared to other adhesion molecules.

Given its dual roles in immune and tumor cells—exhibiting both antitumor and protumor effects—JAML’s therapeutic potential in cancer treatment warrants further investigation. Animal studies suggest that combining agonistic anti-JAML antibodies with PD-1 inhibitors achieves enhanced tumor suppression compared to PD-1 monotherapy. However, significant concerns remain regarding the potential for agonistic anti-JAML antibodies to inadvertently stimulate tumor proliferation in malignancies with high JAML expression. In terms of clinical translation, relevant clinical trials for JAML have not yet been conducted, and research on JAML remains limited to preclinical studies. At the same time, targeted therapy against JAML may lead to off-target effects due to the widespread expression of JAML, potentially affecting the function of normal tissues and cells. For example, it could hinder the physiological migration of inflammatory cells, cause abnormal polarization of macrophages, interfere with intracellular lipid metabolism, and disrupt normal physiological processes, leading to severe drug-related adverse effects. This undoubtedly hinders the clinical translation of JAML, so there is a need to develop tumor-specific JAML antibodies to minimize the risk of off-target effects. To clarify this specificity, it depends on investigating the spatiotemporal expression of JAML in tumor microenvironments.

This review synthesizes current research on JAML, tracing its discovery and elucidating its roles in both tumor and non-tumor tissues, as well as its contributions to various inflammatory diseases and malignancies. By focusing on JAML’s specific overexpression in tumor tissues, we assess its potential as a novel therapeutic target for cancer. In summary, JAML presents significant promise as a therapeutic target with broad clinical applications, offering innovative strategies for advancing antitumor treatments.
